# Proliferation, apoptosis, and fractal dimension analysis for the quantification of intestinal trophism in sole (*Solea solea*) fed mussel meal diets

**DOI:** 10.1186/1746-6148-10-148

**Published:** 2014-07-04

**Authors:** Rubina Sirri, Carlo Bianco, Gionata De Vico, Francesca Carella, Alessio Bonaldo, Giuseppe Sarli, Giada Tondini, Luciana Mandrioli

**Affiliations:** 1 Department of Veterinary Medical Sciences, University of Bologna, Via Tolara di Sopra 50, 40064, Ozzano Emilia, Bologna, Italy; 2 Department of Biological Sciences, Naples University Federico II, Via Mezzocannone, 8, 80134, Naples, Italy

**Keywords:** *Solea solea*, Intestine, Mussel meal, Immunohistochemistry, Image analysis, Cell proliferation, Apoptosis, PCNA, TUNEL, Fractal dimension

## Abstract

**Background:**

The evaluation of intestinal trophism, mainly the mucosal layer, is an important issue in various conditions associated with injury, atrophy, recovery, and healing of the gut. The aim of the present study was to evaluate the kinetics of the proliferation and apoptosis of enterocytes by immunohistochemistry and to assess the complexity of intestinal mucosa by fractal dimension (FD) analysis in *Solea solea* fed different experimental diets.

**Results:**

Histomorphological evaluation of all intestinal segments did not show signs of degeneration or inflammation. Cell proliferation index and FD were significantly reduced with a diet high in mussel meal (MM; p = 0.0034 and p = 0.01063, respectively), while apoptotic index did not show any significant difference for the same comparison (p = 0.3859). Linear regression analysis between apoptotic index (independent variable) and FD (dependent variable) showed a statistically significant inverse relationship (p = 0.002528). Linear regression analysis between cell proliferation index (independent variable) and FD (dependent variable) did not show any significant correlation (p = 0.131582).

**Conclusions:**

The results demonstrated that diets containing increasing levels of mussel meal in substitution of fishmeal did not incite a hyperplastic response of the intestinal mucosa. The mussel meal, which is derived from molluscs, could mimic the characteristics of the sole’s natural prey, being readily digestible, even without increasing the absorptive surface of intestinal mucosa. Interestingly, from this study emerged that FD could be used as a numeric indicator complementary to in situ quantification methods to measure intestinal trophism, in conjunction with functional parameters.

## Background

The evaluation of intestinal trophism, mainly the mucosal layer, is an important issue in various conditions associated with injury, atrophy, recovery, and healing of the gut. The integrity of the mucosal barrier and the speed of either proliferation or apoptosis are other common variables of relevance for many investigative studies [1]. To make these investigations objective and measurable, cellular turnover or morphometric parameters such as villus height, crypt depth, and enterocyte height are frequently used [1]. Focusing on fish, histological analysis of the digestive system is considered a good indicator of the nutritional status of health [2]. The intestine and liver are the most important organs in digestion and the absorption of nutrients from food and, therefore, the monitoring of these organs is considered necessary. Intestinal villi of mammals’ and birds’ digestive systems fit the Euclidean shape (a cylinder-like structure) and allow the employment of the classical morphometric parameters. The morphological peculiarities of the fish intestine that distinguish it from the mammalian intestine, including the lack of distinct crypt compartments and the presence of longitudinal folding of the mucosa instead of villi [3], do not permit the use of this classical approach.

The aim of the present study was to evaluate the kinetics of the proliferation and apoptosis of enterocytes by immunohistochemistry and subsequently, to assess the complexity of intestinal mucosa by fractal dimension analysis in *Solea solea* fed different experimental diets.

## Methods

The experiment was evaluated and approved by the Ethical-Scientific Committee for Animal Experimentation of the University of Bologna, in accordance with the European Community Council directive (86/609/ECC). Fish were obtained from the final sampling of an ongrowing experiment carried out at the Laboratory of Aquaculture, Department of Veterinary Medical Sciences, University of Bologna, Italy. Common sole (*Solea solea*) juveniles used for this experiment were obtained from natural spawning of the broodstock adapted to captivity at the above-mentioned laboratory. From the larval stage, fish were cultured in a 3,000-L circular tank. Larvae were reared using the standard protocol adopted [4,5] and post-larvae and juveniles were fed a commercial diet (Biomar, Denmark, crude protein 63%, total lipids 14%). Before the initiation of the trial, 840 sole juveniles were distributed into twelve 500-L square tanks (bottom surface: 0.64 m2) (70 individuals tank-1) in a completely random manner, to acclimatize them to experimental conditions for 41 days. During this period, fish were fed by automatic feeders for 12 h per day with a mixture of the four experimental diets. The tanks were provided with natural seawater and were connected to a unique closed recirculation system consisting of a mechanical sand filter (0.4 m3 of silica sand, 0.4–0.8 mm, Astral pool PTK 1200 model filter, Servaqua S.A. Barsareny, Spain), an ultraviolet light (PE 25 mJ/cm2: 16 m3 h-1, Blaufish, Barcelona, Spain) and a biofilter (PTK 1200, Astral Pool, Servaqua S.A. Barsareny, Spain). The water exchange rate per tank was 100% every 2 h, whereas the overall water renewal of the system was 5% daily. Temperature was maintained constant at 20 ± 1°C throughout the experiment. The photoperiod was held constant at a 12-h day-length via artificial light (200 lx at the water surface, Delta Ohm luxmeter HD-9221, Delta-Ohm, Padua, Italy). The oxygen level was kept constant (7.5 ± 1.0 mg l-1) by a liquid oxygen system connected to software (B&G Sinergia snc, Chioggia, Italy). Ammonium (total ammonium nitrogen 0.5–0.1 mg L-1), nitrite (NO2- < 0.2 mg L-1) and nitrate (NO3- < 50 mg L-1) were determined spectrophotometrically, daily (Spectroquant Nova 60, Merk, Lab business, Darmstadt, Germany) at 12:00. At the same time, pH (7.8–8.2) and salinity (20 g L-1) were also determined. Each experimental diet was fed to triplicate groups (13.1 ± 2.3 g initial mean body weight), assigned in a completely random manner, over 91 days. Fish were hand-fed twice-daily for 1 h at a time (at 9:00 and 16:00) for 6 days a week and once on Sundays (9:00), to apparent satiation with four isoproteic (53%) and isolipidic (11%) palletized diets containing graded levels of mussel meal (0%, 25%, 50%, and 75%, named respectively MM0, MM25, MM50, and MM75) to replace fish meal (FM). The meal distribution was made following a different tank order daily. To reduce the feeding hierarchies and to allow the access to the feed for all animals, the feed has been distributed evenly over the surface area. At the end of the trial, the average fish weight ± standard deviation (n = 3 tanks, 70 animals in each tank) was 32.4 ± 3.4, 42.4 ± 0.9, 46.0 ± 1.3 and 46.0 ± 3.0 g in the groups fed MM0, MM25, MM50 and MM75, respectively. A linear regression analysis to analyze final weight against the MM dietary inclusion produced a R2 value of 0.71 (p < 0.001).

At the end of the trial, three fish per tank (total of 36 animals) were sampled for gut histology. After euthanasia with a lethal dose of 2-phenoxyethanol, the gut was removed and the intestine was divided into three segments (anterior, intermediate, and posterior); from each segment, a 5 mm-long piece was sectioned and fixed in 10% buffered formalin. Samples were then processed for routine histology to obtain a transversal section, which was stained with haematoxylin and eosin (H&E). Sections were evaluated under a light microscope (Nikon Eclipse 80i, Nikon Corporation, Japan) for degenerative and inflammatory changes.

### Immunohistochemistry

Anti-proliferating cell nuclear antigen (PCNA) antibody was employed for detection of the proliferation rate of enterocytes. Sections were deparaffinised for 30 min in Solvent Plus (Carlo Erba, Reagents S.r.l., Italy) and hydrated in a graded series of alcohols. Subsequently, endogenous peroxidase activity was blocked with 3% hydrogen peroxide in distilled water for 30 min at room temperature. Sections were then treated in citrate buffer (pH 6.0) for 10 min in a microwave oven (750 W) for antigen retrieval. Nonspecific binding was blocked by incubating the sections with 5% normal goat serum and 1% bovine serum albumin (BSA) in PBS buffer (blocking solution) for 1 h at room temperature and then sections were incubated overnight at 4°C with mouse monoclonal PCNA antibody (clone PC10, Cell Signaling Technology, Danvers, MA) diluted 1:4000 in blocking solution. A streptavidin-biotin-peroxidase polymeric complex (SuperPicture kit peroxidise, Zymed® Lab, San Francisco, USA) was used to visualise antibody-antigen interaction and applied on the sections for 10 min at room temperature. The immunohistochemical reaction was developed using a 3,3’-diaminobenzidine (DAB) solution (Sigma-Aldrich Corporate, St. Louis, MO). The slides were counterstained with haematoxylin, dehydrated through alcohol, and cleared in xylene before mounting.

### Terminal deoxynucleotidyl transferase dUTP nick end labelling (TUNEL) assay

The TUNEL method was used to detect the apoptosis rate of enterocytes. The ApopTag® Peroxidase In Situ Apoptosis Detection Kit (Millipore Corporation, Billerica, MA, USA) was employed. Sections were deparaffinised for 30 min in Solvent Plus (Carlo Erba Reagents S.r.l., Italy) and hydrated in a graded series of alcohols. Treatment with proteinase K (20 μg/ml) in PBS for 15 min was performed. Subsequently, endogenous peroxidase activity was blocked with 3% hydrogen peroxide in distilled water for 5 min at room temperature. Equilibration Buffer was then applied on the sections for 3 min and finally, they were incubated with Working Strength TdT Enzyme for 1 h at 37°C in a humid chamber. After washing with Stop/Wash Buffer for 10 min, the secondary antibody anti-digoxigenin peroxidase conjugate was applied on the sections for 30 min at room temperature. After washing, the sections were subjected to the same protocol as used for PCNA.

### Image analysis and cell counting

From each stained section, photomicrographs of five fields per gastrointestinal tract at 20× magnification were captured using a Nikon Digital Sight SD-MS camera (Nikon Corporation, Japan) connected to an optical microscope. Images were then processed using ImageJ 1.46 software, which is freely downloadable [6]. Acquired photographs of PCNA- and TUNEL-stained slides were in 24 bit jpg format, with image resolution of 2560×1920 pixels.

PCNA-positive nuclei of enterocytes were counted using the plugin *color segmentation*[7]. The original image is divided into colour channels, which are manually chosen by the operator as DAB-stained positive nuclei, haematoxylin-stained negative nuclei, cytoplasm of enterocytes, and white background. The software automatically calculates the percentage of area occupied by each chosen colour and, specifically, the percentage of area occupied by PCNA-positive (DAB-stained) nuclei.

In order to eliminate false positivity of non-epithelial cells (erythrocytes, macrophages, lymphocytes, and fibroblasts), the *lamina propria* had been manually erased using the software Adobe Photoshop CS5 (Adobe Systems Inc, San Jose, CA). For apoptotic cell count, the following findings were considered to represent apoptosis [8,9]: (a) marked condensation of chromatin and cytoplasm (apoptotic cells); (b) cytoplasmic fragments with or without condensed chromatin (apoptotic bodies); and (c) intra- and extracellular chromatin (apoptotic micronuclei). TUNEL-positive cells were counted manually in order to eliminate the nonspecific signals due to the application of this method on intestinal sections [10-12], and then the plugin *color segmentation* was employed only to calculate the percentage of area occupied by the other colours (haematoxylin-stained negative nuclei, cytoplasm of enterocytes, and white background).

All data obtained were used to calculate indexes as follows:

$$\mathrm{PCNA}\;\mathrm{index}\;=\frac{\%\;\mathrm{area}\;\mathrm{PCNA}‒\mathrm{positive}\;\mathrm{nuclei}}{\%\;\mathrm{area}\;\mathrm{PCNA}‒\mathrm{positive}\;\mathrm{nuclei}\;+\;\%\;\mathrm{area}\;\mathrm{negative}\;\mathrm{nuclei}}*\;100$$

$$\mathrm{Apoptotic}\;\mathrm{index}\;=\;\frac{n{}^{\circ}\;\mathrm{apoptotic}\;\mathrm{nuclei}/\mathrm{apoptotic}\;\mathrm{bodies}}{n{}^{\circ}\;\mathrm{negative}\;\mathrm{nuclei}\;\mathrm{per}\;\mathrm{field}\;+\;n{}^{\circ}\;\mathrm{apoptotic}\;\mathrm{nuclei}/\mathrm{apoptotic}\;\mathrm{bodies}}*\;100$$

Where the n° of negative nuclei per field is equal to:

$$\frac{\mathrm{total}\;\mathrm{area}\;\mathrm{of}\;\mathrm{the}\;\mathrm{field}\;20x\;*\;\%\;\mathrm{negative}\;\mathrm{nuclei}}{\mathrm{mean}\;\mathrm{area}\;\mathrm{of}\;a\;\mathrm{nucleus}}$$

### Image analysis and fractal dimension (FD) calculation

Eighty photomicrographs of H&E-stained slides of intestinal tracts were obtained in blinded fashion at 4× magnification using a Nikon Digital Sight SD-MS camera connected to an optical microscope. Images were then processed using ImageJ 1.46 software: manual segmentation and thresholding were performed blindly by a second operator to extract the one-pixel wide outline of the intestinal mucosal interface (outline) (Additional file 1). The FD calculation was performed with the Box Counting method using the specific command in ImageJ and setting boxes of 2, 3, 4, 6, 8, 1216, 32, 64, 128, 256, 512, 1024, and 2048 pixels. Image resolution was 2560×1920 pixels and format was 24 bit TIF for the original acquired images of H&E-stained slides; image resolution was 2560×1920 pixels and format was 8 bit Gif for the acquired photographs of outlines.

### Statistical analysis

All data obtained were analysed by the software Statistica 8 (StatSoft Inc., Tulsa, Oklahoma). Normal distribution was tested by Shapiro-Wilk. If normality criteria were met, one way ANOVA with post-hoc Fisher LSD test were run; if normality criteria were not met, Kruskal-Wallis multiple comparison were run. Significance was set as p < 0.05.

## Results

**Figure 1:**
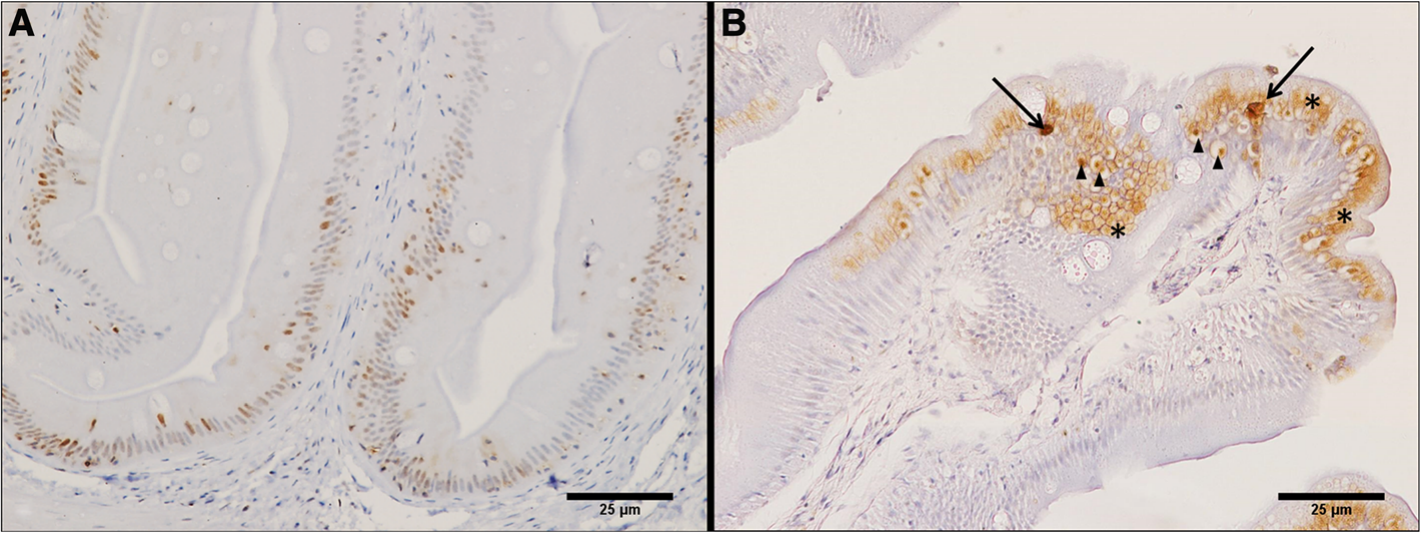
**Immunohistochemistry with anti-PCNA antibody and TUNEL assay. (A)** PCNA-positive nuclei of enterocytes are located mainly in the basal area and along the intestinal folds. **(B)** TUNEL-positive apoptotic cells (arrows) and apoptotic bodies (arrow heads) are located at the apex of intestinal folds. The intrinsic nonspecific binding is also evident (asterisks) (Bars = 25 μm).

**Figure 2:**
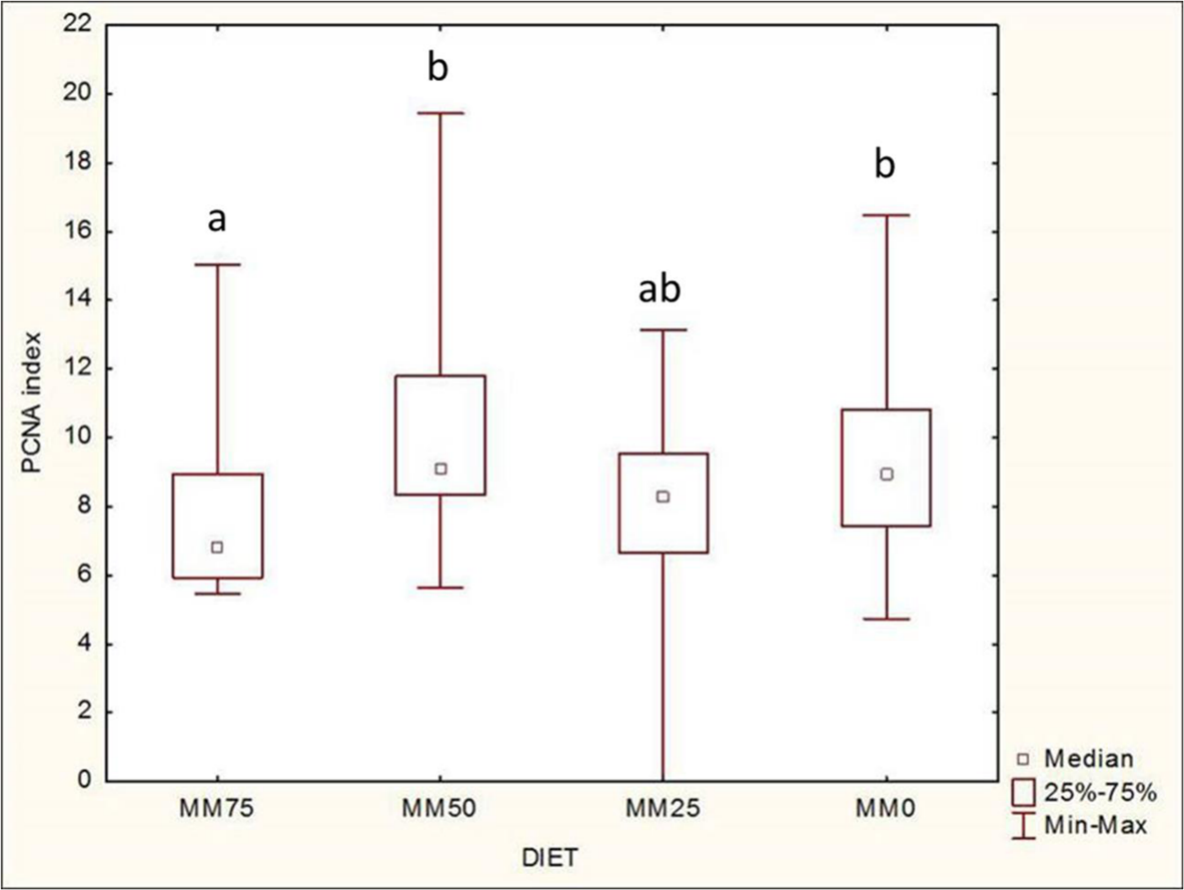
**Diet effect on PCNA index.** Pooled data of PCNA index (9 anterior + 9 intermediate + 9 posterior intestinal tracts = 27 for each group) for each diet displayed PCNA index of MM75 group significantly lower than MM50 and MM0 (Post-hoc Kruskal-Wallis Multiple Comparison n = 108; H = 13.64672, significance p < 0.05) (letters mark significant differences in pairwise comparison).

**Figure 3:**
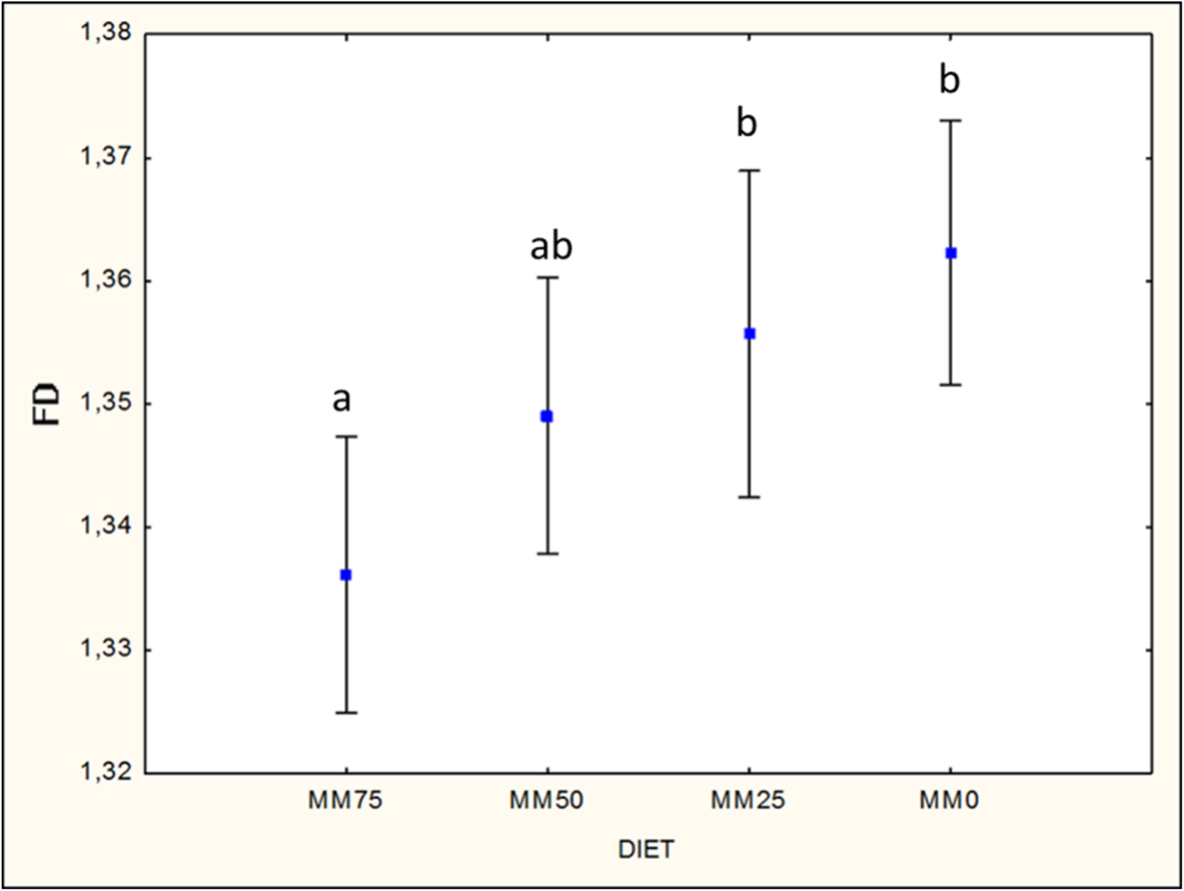
**Diet effect on intestinal fractal dimension.** Pooled data of fractal dimension (respectively, MM0 = 8 anterior + 7 intermediate + 8 posterior intestinal tracts = 23; MM25 = 4 anterior + 5 intermediate + 6 posterior intestinal tracts = 15; MM 50 = 6 anterior + 8 intermediate + 7 posterior intestinal tracts = 21 and MM75 = 6 anterior + 8 intermediate + 7 posterior intestinal = 21) for each diet displayed an increase of FD with the reduction of mussel meal (MM) diet content (Anova Post hoc Fisher LSD test n = 80; mean ± 1.96*SE, significance p < 0.05) (letters mark significant differences in pairwise comparison).

Histomorphological evaluation of all intestinal segments did not show signs of degeneration or inflammation. PCNA labelling revealed a brown, intense, and homogeneous nuclear staining of enterocytes mainly in the basal area and along the intestinal folds (Figure 1A). TUNEL labelling revealed a brown, intense staining of scattered single condensed nuclei or nuclear fragments (Figure 1B).PCNA index was not normally distributed (n = 108; SW W = 0.96290; p = 0.00418). PCNA index in enterocytes showed a significant decrease in parallel with the increase in MM (n = 108; H = 13.64672; p = 0.0034) (Figure 2). With respect to the intestinal tract, PCNA index did not show significant differences (n = 108; H = 4.298455; p = 0.1166). Apoptotic index was not normally distributed (n = 108; SW W = 0.45922; p = 0.00000) and did not show significant differences with respect to the diets (n = 108; H = 3.037620; p = 0.3859). With respect to the intestinal tract, apoptotic index was significantly higher in the posterior segment compared with the anterior and intermediate segments (n = 108; H = 21.21437; p = 0.0000). FD was normally distributed (n = 80; Shapiro-Wilk W = 0.97847; p = 0.19599) and showed a significant decrease with a diet that was high in MM (n = 80; F(3.76) = 3.9997; p = 0.01063) (Figure 3). With respect to the intestinal tract, FD did not show significant differences (n = 80; current effect: F(2.77) = 1.3182; p = 0.27359). Linear regression analysis between apoptotic index (independent variable) and FD (dependent variable) showed a statistically significant inverse relationship (Beta = -0.337225; p = 0.002528). Linear regression analysis between PCNA index (independent variable) and FD (dependent variable) did not show a significant correlation (Beta = 0.164612; p = 0.131582).

## Discussion

In the present study, the histomorphological evaluation of all three intestinal segments did not show signs of degeneration or inflammation, suggesting that MM could be considered a valid substitute for FM in the diet of sole. Immunohistochemistry with PCNA antibody and the TUNEL method have been widely used in fish species to demonstrate the renewal of enterocytes in studies ranging from experimental diet to environmental and toxicological assessments [13-21]. Focusing on the TUNEL method for apoptosis assessment, it has been widely demonstrated that it can produce an intrinsic nonspecific labelling. This does not represent a background staining but a binding with DNA fragments produced by endogenous cytoplasmic endonuclease that is physiologically present, or by antigen retrieval pretreatment [10-12,22,23]. In the present study, the non-specific TUNEL labelling was cytoplasmic, patchy, and hazy, with intense peripheral membranous positivity. Despite these apparent limitations, the TUNEL method coupled to H&E-stained sections enabled us to univocally identify cells undergoing apoptosis [12].

PCNA index showed a decrease in enterocytes proliferation with a diet with major inclusion of MM (MM75) with respect to the control diet (MM0), supporting the hypothesis of an hyperplastic adaptive response induced by a diet that deviates from the feeding habits of this species (a benthonic organism). On the other hand, apoptotic index was not influenced by the diet, probably because dietetic changes were not sufficient to induce a pro-apoptotic response compared with xenobiotics or hormonal stimuli [13,14].

Moreover, the results obtained demonstrated a relationship between reduced enterocytes proliferation and lessened complexity of mucosal folding, expressed by a lower FD, in the diet with the highest MM content. The biological interpretation of this result can be that diets containing high MM (MM75) are more similar to the natural feeding of sole. On the contrary, the control diet (MM0), which is usually administered in this reared species, is probably far from an optimal formulation; this could have triggered an hyperplastic adaptive response of the intestinal mucosa, supported by a higher proliferation index, with the aim of increasing the absorptive surface. In linear regression analysis, an inverse and significant correlation between FD and apoptosis index emerged, confirming that a higher apoptosis rate corresponded to more simple mucosal folding even if there was no significant difference in apoptosis index in relation to the diet.

This study demonstrated the usefulness of FD calculation as an alternative method to immunohistochemistry for the detection of intestinal trophism; FD permits a more rapid assessment of histological sections, reducing the costs of reagents and time.

The FD calculation is able to transduce shape complexity in analytical quantitative data, harmonising the gap between structural features (typically qualitative) and functional quantitative measures. The fractal theory has been widely used in physics, biology, and medical fields and particularly in histology and histopathology. The FD of a pathological image can be interpreted as a snapshot of an evolving process; it is usually fractional and increases with the object’s complexity [24,25]. In Euclidean shapes, a straight line has FD 1, whereas a fractal curve has a value of between 1 and 2 [26-28]. What the FD parameter provides is a statistical estimate of how an object fills a space across a broad range of scales; it is a useful parameter for the characterisation of complex, irregular structures, the analysis of which, when examined mathematically, denotes figures that resemble themselves when examined on different size scales (self-similarity) [29]. Focusing on histopathology, computing FD captures the architectural complexity of the histology specimen [27,30]. To the best of our knowledge, there have been no literature reports investigating FD calculation of the intestine. The originality of the proposed method resides in the substitution or integration of the conventional method for the detection of cell kinetics with a method of assessment of the complexity of the mucosal lining, thus intestinal trophism.

## Conclusions

The results demonstrated that diets containing increasing levels of mussel meal in substitution of fishmeal did not incite a hyperplastic response of the intestinal mucosa. Fishes are not commonly present in the natural diet of sole, whose natural trophic profile is mainly composed of polychaetes and molluscs, as well as crustaceans. The mussel meal, which is derived from molluscs, could mimic the characteristics of the sole’s natural prey, being readily digestible, even without increasing the absorptive surface of intestinal mucosa. Interestingly, from this study emerged that FD could be used as a numeric indicator complementary to in situ quantification methods to measure intestinal trophism, in conjunction with functional parameters.

## Abbreviations

H&E: Haematoxylin and eosin; FD: Fractal dimension; PCNA: Proliferating cell nuclear antigen; MM: Mussel meal; FM: Fish meal; BSA: Bovine serum albumin; PBS: Phosphate-buffered saline; TUNEL: Terminal deoxynucleotidyl transferase dUTP nick end labelling; DAB: 3,3’-diaminobenzidine.

## Competing interests

The authors declare that they have no competing interests.

## Authors’ contributions

RS performed the immunohistochemistry and drafted the manuscript. CB performed fractal dimension analysis and statistics. GDV helped in the application of fractal dimension. FC helped in capturing histological photos. AB participated in the zootechnical design and coordination. GS participated in the study design and critically revised the manuscript. GT helped in image analysis and capturing immunohistochemical photos. LM performed image analysis and drafted the manuscript. All authors read and approved the final manuscript.

## Supplementary Material

Additional file 1 Example of image processing and Box Counting FD calculation of an intestinal segment. Click here for file
